# Recent Advances in Tracking Devices for Biomedical Ultrasound Imaging Applications

**DOI:** 10.3390/mi13111855

**Published:** 2022-10-29

**Authors:** Chang Peng, Qianqian Cai, Mengyue Chen, Xiaoning Jiang

**Affiliations:** 1 School of Biomedical Engineering, ShanghaiTech University, Shanghai 201210, China; 2 Department of Mechanical and Aerospace Engineering, North Carolina State University, Raleigh, NC 27695, USA

**Keywords:** tracking, ultrasound imaging, optical tracking, electromagnetic tracking, 3D ultrasound imaging, ultrasound–guided interventions, ultrasound image fusion

## Abstract

With the rapid advancement of tracking technologies, the applications of tracking systems in ultrasound imaging have expanded across a wide range of fields. In this review article, we discuss the basic tracking principles, system components, performance analyses, as well as the main sources of error for popular tracking technologies that are utilized in ultrasound imaging. In light of the growing demand for object tracking, this article explores both the potential and challenges associated with different tracking technologies applied to various ultrasound imaging applications, including freehand 3D ultrasound imaging, ultrasound image fusion, ultrasound-guided intervention and treatment. Recent development in tracking technology has led to increased accuracy and intuitiveness of ultrasound imaging and navigation with less reliance on operator skills, thereby benefiting the medical diagnosis and treatment. Although commercially available tracking systems are capable of achieving sub-millimeter resolution for positional tracking and sub-degree resolution for orientational tracking, such systems are subject to a number of disadvantages, including high costs and time-consuming calibration procedures. While some emerging tracking technologies are still in the research stage, their potentials have been demonstrated in terms of the compactness, light weight, and easy integration with existing standard or portable ultrasound machines.

## 1. Introduction

An object tracking system locates a moving object (or multiple objects) through time and space [1]. The main aim of a tracking system is to identify an object regarding the position and orientation in space recorded in an extension of time, characterized by precision, accuracy, working range as well as degree-of-freedom (DOF), depending on the systems and applications [2]. With the rapid development of computational and sensing technologies, nowadays tracking systems have been widely utilized in various fields, including robotics [3,4], military [5,6], medicine [7,8] and sports [9,10]. In the medical field, tracking of rotation and translation of medical instruments or patients plays a substantial role in many important applications, such as diagnostic imaging [11], image-guided navigation systems for intervention and therapy [12,13], as well as rehabilitation medicine [14].

Ultrasound (US) imaging is a well-established imaging modality that has been widely utilized in clinical practice for diagnosing diseases or guiding decision-making in therapy [15]. Compared with other medical imaging modalities, such as computed tomography (CT) and magnetic resonance imaging (MRI), US shows the major advantages of real-time imaging, non-radiation exposure, low-cost, and ease to apply [16]. Despite its many advantages, ultrasonography is considered to be highly operator-dependent [17]. Manually guiding of the US probe to obtain reproducible image acquisition is challenging. Moreover, in order to correctly interpret the information acquired by the scanning, rich clinical experience is required for sonographers. Besides the operator dependency that brings the high risk of interpretive error influencing the diagnosis and therapy results, the restricted field of view (FOV) of US probe poses challenges for image visualization and feature localization, thus limiting diagnosis or therapy accuracy. The integration of object tracking system with US imaging can resolve the above-mentioned limitations. By integrating tracking devices with US probes, an extended FOV of US probe can be obtained, resulting in a less operator-dependent scanning procedure and more accurate results. Over the past decade, there has been a significant growth of studies on integration of various tracking systems with US imaging systems for biomedical and healthcare applications. The applying of emerging tracking systems for biomedical US imaging applications has resulted in improved accuracy and intuitiveness of US imaging and navigation with less reliance on operator skills, thereby benefiting the medical diagnosis and therapy.

**Figure 1:**
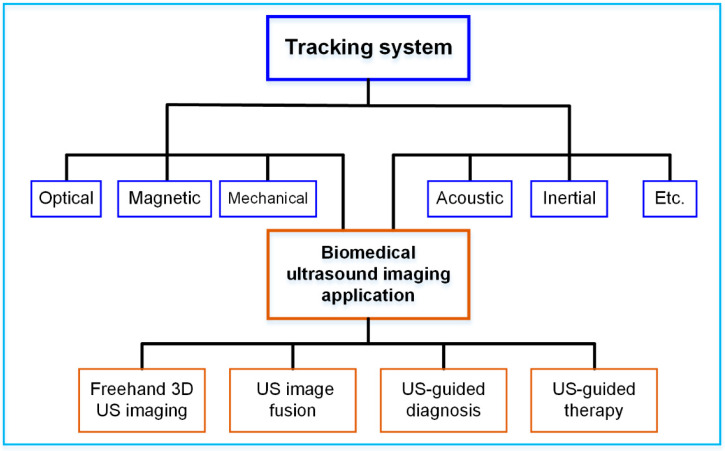
Various tracking systems for biomedical US imaging applications.

The purpose of this article is to provide a literature review on the various tracking systems for biomedical US imaging applications, as illustrated in Figure 1. The rest of the article is organized as follows: in Section 2, the principles of different tracking techniques, including optical tracking, electromagnetic tracking, mechanical tracking, acoustic tracking and inertial tracking, are summarized. The typical tracking systems and their technical performances, such as accuracy and latency are provided in Section 3. Section 4 details the advancement of different tracking systems for US imaging applications, including freehand 3D US imaging, US image fusion, US-guided diagnosis, and US-guided therapy. Finally, a summary and concluding remarks are presented in Section 5.

## 2. Physical Principles of Tracking Technologies

The latest advancements in tracking technologies have enabled conventional medical devices to be equipped with more advanced functions. In biomedical US imaging, object tracking technologies are key to locate US probes and other medical tools for precise operation and intuitive visualization. The underlying physical principles behind the most common tracking technologies will be reviewed in this section.

### 2.1. Optical Tracking

An optical tracking system is among the most precise tracking technologies with 6 DOF that achieves a sub-millimeter accuracy level. Multiple spatially synchronized cameras track the markers attached to the target in the designed space. There are two types of markers: active and passive [18]. Infrared light emitting diodes (LEDs) are used in active markers for the purpose of emitting invisible light that can be detected by cameras. A passive marker is covered with a retro-reflective surface that can reflect incoming infrared light back to the camera. There are usually three or more unsymmetrical markers in a target object. The 6 DOF position and orientation of the object are determined by triangulation [19].

**Figure 2:**
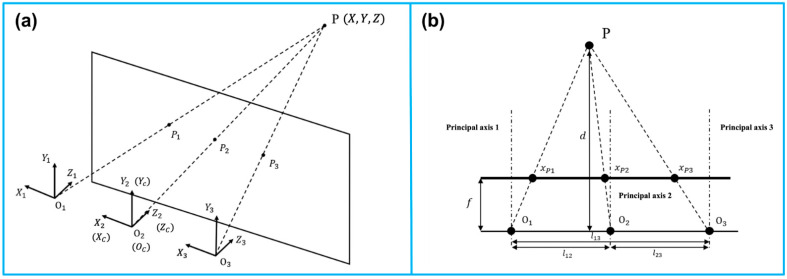
Principle of the camera-based optical tracking technique. (**a**) The three coordinates represent the three lenses integrated in the camera bar. The camera bar is looking at point *P*(*x*, *y*, *z*), where *P*_1_, *P*_2_, and *P*_3_ are the intersections on the image plane. (**b**) The top view demonstrates the similar triangles used to calculate the position information. The depth *d* of point *P* can be determined via triangulation. Reprinted from [20] with permission.

Similar to the human vision system, optical tracking requires at least two cameras that are fixed at a known distance from each other. Adding additional cameras will improve tracking accuracy and robustness. In Figure 2, a trinocular vision system is illustrated as an example of triangulation [20]. P is observed simultaneously by three lenses. Additionally, it generates three projection points on the focal plane. A right-handed, Y-up coordinate is assigned to each lens, with coordinate origins at $O_{1}$, $O_{2}$, and $O_{3}$. The reference coordinate $X_{c}Y_{c}Z_{c}$ coincides with the coordinate $X_{2}Y_{2}Z_{2}$ of the middle lens. A baseline $l_{ij}$ is defined to be the distance between any two lenses. Three parallel principal axes are present in the three fixed lenses. There is a perpendicular relationship between the principal axes and the baseline.

To perceive the depth with two lenses i and j, where i, j∈ {1, 2, 3}, and i ≠ j. With the known focal length f, and the disparity $x_{pi}-x_{pj}$ representing the offset between the two projections in the XOZ plane, the depth Z can be derived as

(1) $$Z=\frac{fl_{ij}}{x_{pi}-x_{pj}}$$

Furthermore, the other two coordinates of P(X,Y,Z) can thus be calculated as

(2) $$X=\frac{x_{pi}Z}{f}\;Y=\frac{y_{pj}Z}{f}$$

Given all the markers’ positions, the orientation of the marker set is determined. With the known positions of all markers, the orientation of the target is also determined. The 6 DOF poser information is delivered in the form of a transformation matrix $T_{M}^{C}$, with the subscript and superscript representing the marker set coordinate (M) to the camera coordinate (C), respectively.

(3) $$T_{M}^{C}=\left[\begin{array}{cc} R & p\\ 0 & 1 \end{array}\right]$$

where $p={\left(X_{M},Y_{M},Z_{M}\right)}^{T}$ is the offset between the two origins of coordinates M and C. Additionally, R is a 3×3 rotational matrix in the form of

(4) $$R=\left[\begin{array}{ccc} cos\varphi cos\theta & cos\varphi sin\theta sin\psi -sin\varphi cos\psi & cos\varphi sin\theta cos\psi +sin\varphi sin\psi \\ sin\varphi cos\theta & sin\varphi sin\theta sin\psi +cos\varphi cos\psi & sin\varphi sin\theta cos\psi -cos\varphi sin\psi \\ -sin\theta & cos\theta sin\psi & cos\theta cos\psi \end{array}\right]$$

From the above matrix, the orientations φ (yaw), θ (pitch), and ψ (roll) can thus be solved as follows:

(5) $$\{\begin{array}{c} \varphi =arctan\frac{R_{12}}{R_{11}}\\ \theta =-arcsinR_{31}\\ \psi =arctan\frac{R_{32}}{R_{33}} \end{array}$$

### 2.2. Electromagnetic Tracking

Tracking systems using electromagnetic signals can also provide sub-millimeter accuracy in dynamic and real-time 6 DOF tracking. Its advantages include being lightweight and free of line-of-sight. In biomedical engineering, it is commonly used for the navigation of medical tools.

Electromagnetic tracking systems consist of four modules: transmission circuits, receiving circuits, digital signal processing units, and microcontrollers. Based on Faraday’s law, electromagnetic tracking systems use transmitted voltages to estimate the position and orientation of objects in alternating magnetic fields when the object is coupled to a receiver sensor [21,22].

**Figure 3:**
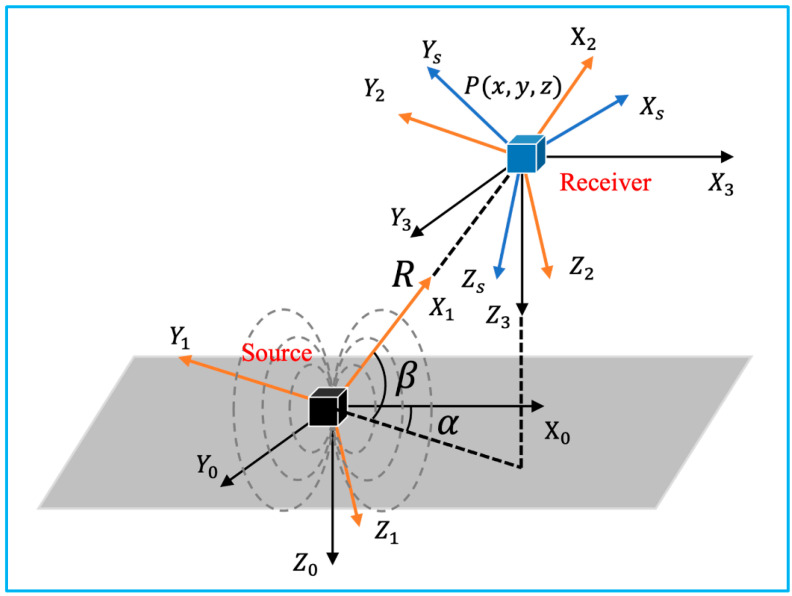
Coordinate systems involved in the 3D electromagnetic tracking (adapted from [21]).

Figure 3 illustrates the involved coordinate systems. The reference coordinate system is denoted as $X_{0}Y_{0}Z_{0}$, which is fixed at the emission coil. $X_{s}Y_{s}Z_{s}$ is the coordinate system fixed at the receiver sensor. The location of its origin $O_{1}(X,Y,Z)$ with respect to $X_{0}Y_{0}Z_{0}$, can also be denoted as $O_{1}\left(R,\alpha ,\beta \right)$ in spherical coordinate. Additionally, the orientation is represented as Euler angles φ, θ, and ψ.

Assuming the excitation current $i\left(t\right)=I_{i}\sin\left(\omega t+\phi \right)$, the transmitter parameter along each direction defined as $C_{i}=\frac{{\mu N}_{i}I_{i}S_{i}}{4\pi }$, where i=x, y, z, $N_{i}$ is the number of turns in the coil, and $S_{i}$ is the area of the coil, the excitation signal can be defined as

(6) $$f_{0}=C=\left[\begin{array}{ccc} C_{x} & 0 & 0\\ 0 & C_{y} & 0\\ 0 & 0 & C_{z} \end{array}\right]$$

Accordingly, the receiver parameter can be written as

(7) $$K=\left[\begin{array}{ccc} K_{x} & 0 & 0\\ 0 & K_{y} & 0\\ 0 & 0 & K_{z} \end{array}\right]$$

where $k_{i}={\omega n}_{i}g_{i}s_{i}$, with ω representing the radian frequency of the source excitation signal, $n_{i}$ and $s_{i}$ denoting the number of turns in the coil and the area of the coil, and $g_{i}$ indicating the system gain. According to Faraday’s law of induction, the amplitude of the voltage from the receiver coil is expressed as

(8) $$S_{ij}=\omega K_{j}C_{i}h\left(R,\alpha ,\beta ,\varphi ,\theta ,\psi \right)=\omega K_{j}B_{ij}$$

With the position and orientation of the receiver fixed, the value of h(R,α,β,φ,θ,ψ) is determined. $B_{\mathrm{ij}}$ is the amplitude of the magnetic field produced at that location.

From Equation (8), by defining the final sensor output to be

(9) $$f_{s}=\left[\begin{array}{ccc} s_{\mathrm{xx}} & s_{\mathrm{yx}} & s_{\mathrm{zx}}\\ s_{\mathrm{xy}} & s_{\mathrm{yy}} & s_{\mathrm{zy}}\\ s_{\mathrm{xz}} & s_{\mathrm{yz}} & s_{\mathrm{zz}} \end{array}\right]$$

the magnetic field expressed in $X_{s}Y_{s}Z_{s}$ is

(10) $$B=K^{-1}f_{s}=\left[\begin{array}{ccc} \frac{s_{\mathrm{xx}}}{k_{x}} & \frac{s_{\mathrm{xy}}}{k_{y}} & \frac{s_{\mathrm{xz}}}{k_{x}}\\ \frac{s_{\mathrm{yx}}}{k_{y}} & \frac{s_{\mathrm{yy}}}{k_{y}} & \frac{s_{\mathrm{yz}}}{k_{y}}\\ \frac{s_{\mathrm{zx}}}{k_{z}} & \frac{s_{\mathrm{zy}}}{k_{z}} & \frac{s_{\mathrm{zz}}}{k_{z}} \end{array}\right]$$

When the equivalent transmitter coil along each direction is excited, the square amplitude of the magnetic field P can be expressed as

(11) $$P=\left[\begin{array}{c} {\frac{s_{\mathrm{xx}}}{k_{x}}}^{2}+{\frac{s_{\mathrm{yx}}}{k_{x}}}^{2}+{\frac{s_{\mathrm{zx}}}{k_{x}}}^{2}\\ {\frac{s_{\mathrm{xy}}}{k_{y}}}^{2}+{\frac{s_{\mathrm{yy}}}{k_{y}}}^{2}+{\frac{s_{\mathrm{zy}}}{k_{y}}}^{2}\\ {\frac{s_{\mathrm{xz}}}{k_{z}}}^{2}+{\frac{s_{\mathrm{yz}}}{k_{z}}}^{2}+{\frac{s_{\mathrm{zz}}}{k_{z}}}^{2} \end{array}\right]=\left[\begin{array}{c} \frac{4C_{x}^{2}}{r^{6}}\left(x^{2}+\frac{1}{4}y^{2}+\frac{1}{4}z^{2}\right)\\ \frac{4C_{y}^{2}}{r^{6}}\left(\frac{1}{4}x^{2}+y^{2}+\frac{1}{4}z^{2}\right)\\ \frac{4C_{z}^{2}}{r^{6}}\left(\frac{1}{4}x^{2}+\frac{1}{4}y^{2}+z^{2}\right) \end{array}\right]$$

Canceling out the unknown position (x, y, z) by summing up all three entries, the only unknown parameter r can be deduced.

With two rotational matrices T(α) and T(β) as

(12) $$T\left(\alpha \right)=\left[\begin{array}{ccc} cos\alpha & sin\alpha & 0\\ -sin\alpha & cos\alpha & 0\\ 0 & 0 & 1 \end{array}\right]\;T\left(\beta \right)=\left[\begin{array}{ccc} cos\beta & 0 & -sin\beta \\ 0 & 1 & 0\\ sin\beta & 0 & cos\beta \end{array}\right]$$

A key matrix F can be defined as

(13) $$\;\;\;\;\;\;\;\;\;\;\;\;\;\;\;\;\;\;\;\;\;\;\;\;\;\;\;\;\;\begin{array}{cc} F & =r^{6}{\left(f_{0}^{T}\right)}^{-1}f_{s}^{T}K^{-1}K^{-1}f_{s}f_{0}^{-1}\\ & =\left[\begin{array}{ccc} 1+3\cos^{2}{\alpha \cos}^{2}\beta & 3{\sin\alpha \cos\alpha \cos}^{2}\beta & -3\cos\alpha \sin\beta \cos\beta \\ 3{\sin\alpha \cos\alpha \cos}^{2}\beta & 1+3\sin^{2}{\alpha \cos}^{2}\alpha & -3\sin\alpha \sin\beta \cos\beta \\ -3\cos\alpha \sin\beta \cos\beta & -3\sin\alpha \sin\beta \cos\beta & 1+3\sin^{2}\beta \end{array}\right] \end{array}$$

As $s_{ij}$, ω, $C_{i}$, $K_{j}$ as known parameters, the unknown α and β can be solved as

(14) $$\{\begin{array}{l} \alpha =arctan\frac{F_{23}}{F_{13}}\;\;\;\;\;\;\;\;\;\;\;\\ \beta =arcsin\sqrt{\frac{F_{33}-1}{3}} \end{array}$$

From the spherical coordinates, the position of the target *P* can be written as

(15) $$\{\begin{array}{c} x=rcos\beta cos\alpha \\ y=rcos\beta sin\alpha \\ z=rsin\beta \;\;\;\;\;\;\;\;\; \end{array}\;$$

Substituting

(16) $$T\left(\varphi \right)=\left[\begin{array}{ccc} 1 & 0 & 0\\ 0 & cos\varphi & sin\varphi \\ 0 & -sin\varphi & cos\varphi \end{array}\right]\;T\left(\theta \right)=\left[\begin{array}{ccc} cos\theta & 0 & -sin\theta \\ 0 & 1 & 0\\ sin\theta & 0 & cos\theta \end{array}\right]\;T\left(\psi \right)=\left[\begin{array}{ccc} cos\psi & sin\psi & 0\\ -sin\psi & cos\psi & 0\\ 0 & 0 & 1 \end{array}\right]$$

into

(17) $$f_{s}=KB=\frac{2}{r^{3}}KT\left(\varphi \right)T\left(\theta \right)T\left(\psi \right)T\left(-\alpha \right)T\left(-\beta \right)ST\left(\beta \right)T\left(\alpha \right)f_{0}$$

matrix T is defined as

(18) $$\begin{array}{cc} T & =T\left(\varphi \right)T\left(\theta \right)T\left(\psi \right)\\ = & \left[\begin{array}{ccc} \cos\varphi \cos\psi & \cos\varphi \sin\psi & -\sin\varphi \\ \sin\theta \sin\varphi \cos\psi -\cos\theta \sin\psi & \sin\varphi \sin\theta \sin\psi +\cos\theta \cos\psi & \sin\theta \cos\varphi \\ \cos\theta \sin\varphi \cos\psi +\sin\theta \sin\psi & \cos\theta \sin\varphi \sin\psi +\sin\theta \cos\psi & \cos\theta \cos\varphi \end{array}\right] \end{array}$$

Therefore, the target’s orientation can be solved as

(19) $$\{\begin{array}{l} \varphi =arctan\left(-\frac{T_{13}}{{\left(T_{23}^{2}+T_{33}^{2}\right)}^{\frac{1}{2}}}\right)\\ \theta =arctan\left(\frac{T_{23}}{T_{33}}\right)\;\;\;\;\;\;\;\;\;\;\;\;\;\;\;\;\;\;\;\;\;\;\\ \psi =arctan\left(\frac{T_{12}}{T_{11}}\right)\;\;\;\;\;\;\;\;\;\;\;\;\;\;\;\;\;\;\;\;\;\;\; \end{array}$$

### 2.3. Mechanical Tracking

**Figure 4:**
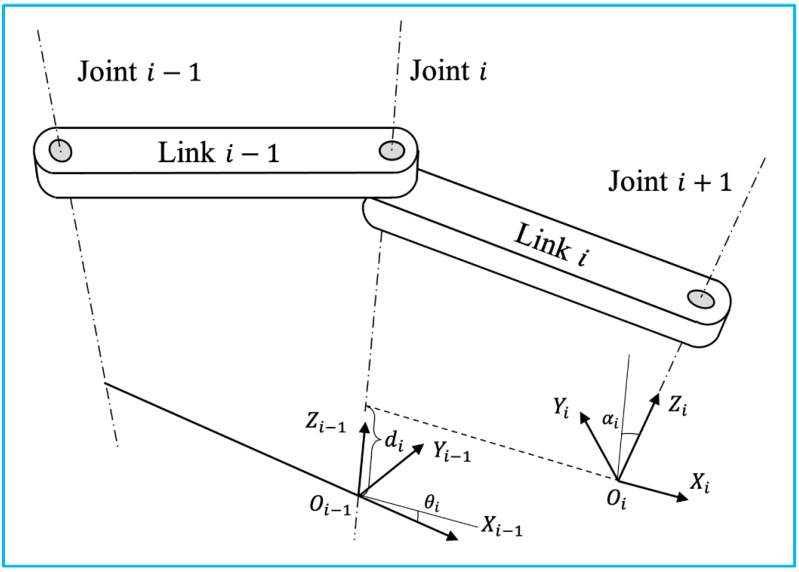
Two adjacent links with a revolute joint with the relative position denoted using Denavit-Hartenberg convention (adapted from [23]).

Robotic tracking systems use articulated robotic arms to manipulate the target attached to the end effector. Typically, industrial robots are composed of a number of joints and links. Joint movement is continuously detected by potentiometers and encoders in-stalled on each joint. The real-time position and orientation of the effector can be deter-mined by calculating homogeneous transformations from the collected robotic dynamics. In clinical practice, the operator can either control the movement of the robot to a certain location with the desired orientation, or specify the destination, and the robot solves the path using inverse dynamics based on the spatial information of the destination and the architecture of the robot. Following the Denavit and Hartenberg notation, the forward dynamics will be applied to illustrate how the 6 DOF pose information is transformed be-tween adjacent joints and links, as illustrated in Figure 4 [23].

The 4×4 homogeneous transformation matrix for each step is shown below.

(20) $$A_{1}=\left[\begin{array}{cccc} cos\theta_{i} & -sin\theta_{i} & 0 & 0\\ sin\theta_{i} & cos\theta_{i} & 0 & 0\\ 0 & 0 & 1 & 0\\ 0 & 0 & 0 & 1 \end{array}\right],\;A_{2}=\left[\begin{array}{cccc} 1 & 0 & 0 & a_{i}\\ 0 & 1 & 0 & 0\\ 0 & 0 & 1 & 0\\ 0 & 0 & 0 & 1 \end{array}\right]\;A_{3}=\left[\begin{array}{cccc} 1 & 0 & 0 & 0\\ 0 & 1 & 0 & 0\\ 0 & 0 & 1 & d_{i}\\ 0 & 0 & 0 & 1 \end{array}\right],\;A_{1}=\left[\begin{array}{cccc} 1 & 0 & 0 & 0\\ 0 & cos\alpha_{i} & -sin\alpha_{i} & 0\\ 0 & sin\alpha_{i} & cos\alpha_{i} & 0\\ 0 & 0 & 0 & 1 \end{array}\right]$$

(21) $$\left[\begin{array}{c} x_{i}\\ y_{i}\\ z_{i}\\ 1 \end{array}\right]=\underset{T}{\underbrace{A_{1}A_{2}A_{3}A_{4}}}\left[\begin{array}{c} x_{i}\\ y_{i}\\ z_{i}\\ 1 \end{array}\right]=\left[\begin{array}{cccc} cos\theta_{i} & -sin\theta_{i}cos\alpha_{i} & sin\theta_{i}sin\alpha_{i} & a_{i}cos\theta_{i}\\ sin\theta_{i} & cos\theta_{i}cos\alpha_{i} & -cos\theta_{i}sin\alpha_{i} & a_{i}sin\theta_{i}\\ 0 & sin\alpha_{i} & cos\alpha_{i} & d_{i}\\ 0 & 0 & 0 & 1 \end{array}\right]\left[\begin{array}{c} x_{i}\\ y_{i}\\ z_{i}\\ 1 \end{array}\right]$$

The transformation matrix T can also be represented in terms of position $\left(p_{x},p_{y},p_{z}\right)$ in the reference coordinate and orientation (φ,θ,ψ) in yaw-pitch-roll representation.

(22) $$T=\left[\begin{array}{cccc} cos\varphi cos\theta & cos\varphi sin\theta sin\psi -sin\varphi cos\psi & cos\varphi sin\theta cos\psi +sin\varphi sin\psi & p_{x}\\ sin\varphi cos\theta & sin\varphi sin\theta sin\psi +cos\varphi cos\psi & sin\varphi sin\theta cos\psi -cos\varphi sin\psi & p_{y}\\ -sin\theta & cos\theta sin\psi & cos\theta cos\psi & p_{z}\\ 0 & 0 & 0 & 1 \end{array}\right]$$

Based on Equations (21) and (22), the 6 DOF pose information can be solved as

(23) $$\{\begin{array}{c} p_{x}=a_{i}cos\theta_{i}\\ p_{y}=a_{i}sin\theta_{i}\\ p_{z}=d_{i}\;\;\;\;\;\;\;\; \end{array}$$

(24) $$\{\begin{array}{c} \theta =arcsin\left(-T_{31}\right)\\ \varphi =arccos\left(\frac{T_{33}}{cos\theta }\right)\\ \psi =arccos\left(\frac{T_{11}}{cos\theta }\right) \end{array}$$

### 2.4. Acoustic Tracking

**Figure 5:**
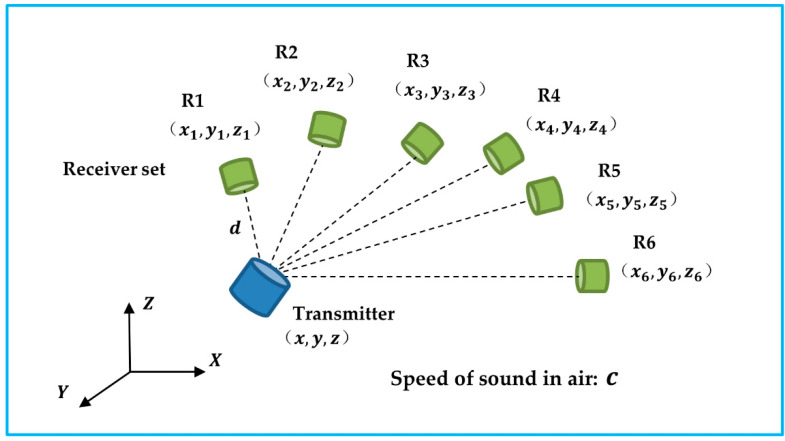
The transmitter sends out carrier signals to the receiver set. The unknow transmitter’s position and the speed of sound can be solved based of the time difference of arrival.

An acoustic tracking system is one of the three DOF positional tracking systems. To determine the spatial location of the target object, an ultrasonic transmitter transmits a carrier signal that is received by multiple receivers operating at the same frequency. Specifically, by estimating the actual travel/arrival times (TOF/TOA) or the time difference between travel/arrival (TDOF/TDOA), the 3D coordinates of the object (x, y, z) can be determined to centimeter accuracy levels with receivers fixed at known locations, as shown in Figure 5. A TDOF/TDOA algorithm is more practical and accurate than a TOF/TOA algorithm since it circumvents the synchronization issue between the transmitter and receiver. A limitation of acoustic tracking is that the accuracy of the tracking is affected by the temperature and air turbulence in the environment [24]. This problem can be addressed by including the speed of sound (*c*) as an unknown parameter in the calculation [25].

The predetermined geometry of the receivers was notated as $\left(x_{i},y_{i},z_{i}\right)$, where i represents the ith receiver, where i {1, 2, 3, 4, 5, 6}. The reference distance between the transmitter and receiver R1 is denoted as d. $\Delta T_{1j}$ indicates the TDOF between receiver R1 and Rj, where j∈ {2, 3, 4, 5, 6}.

(25) $$\left[\begin{array}{ccccc} 2x_{1}-2x_{2} & 2y_{1}-2y_{2} & 2z_{1}-2z_{2} & -2\Delta T_{12} & -2\Delta T_{12}^{2}\\ 2x_{1}-2x_{3} & 2y_{1}-2y_{3} & 2z_{1}-2z_{3} & -2\Delta T_{13} & -2\Delta T_{13}^{2}\\ 2x_{1}-2x_{4} & 2y_{1}-2y_{4} & 2z_{1}-2z_{4} & -2\Delta T_{14} & -2\Delta T_{14}^{2}\\ 2x_{1}-2x_{5} & 2y_{1}-2y_{5} & 2z_{1}-2z_{5} & -2\Delta T_{15} & -2\Delta T_{15}^{2}\\ 2x_{1}-2x_{6} & 2y_{1}-2y_{6} & 2z_{1}-2z_{6} & -2\Delta T_{16} & -2\Delta T_{16}^{2} \end{array}\right]\left[\begin{array}{c} x\\ y\\ z\\ cd\\ c^{2} \end{array}\right]\;=\left[\begin{array}{c} x_{1}^{2}+y_{1}^{2}+z_{1}^{2}-x_{2}^{2}-y_{2}^{2}-z_{2}^{2}\\ x_{1}^{2}+y_{1}^{2}+z_{1}^{2}-x_{3}^{2}-y_{3}^{2}-z_{3}^{2}\\ x_{1}^{2}+y_{1}^{2}+z_{1}^{2}-x_{4}^{2}-y_{4}^{2}-z_{4}^{2}\\ x_{1}^{2}+y_{1}^{2}+z_{1}^{2}-x_{5}^{2}-y_{5}^{2}-z_{5}^{2}\\ x_{1}^{2}+y_{1}^{2}+z_{1}^{2}-x_{6}^{2}-y_{6}^{2}-z_{6}^{2} \end{array}\right]$$

Occlusion can also affect the accuracy of an acoustic tracking system. A receiver configuration should be taken into serious consideration when implementing such a system for biomedical US imaging applications [26]. In addition to reducing occlusion, an optimal configuration also contributes to improved tracking performance. As an acoustic tracking system is not able to identify a target’s object, other tracking systems, such as inertial tracking, are frequently required [27].

### 2.5. Inertial Tracking

**Figure 6:**
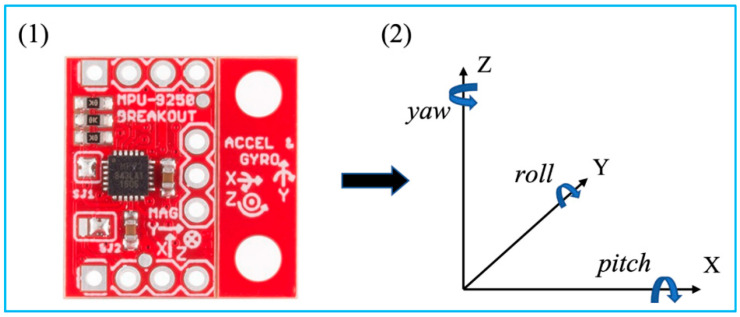
MPU 9250 and its coordinate system with Euler angles. Reprinted from [27] with permission.

Inertial tracking systems are based on an inertial measurement unit (IMU), which is a small, lightweight, cost-effective sensor enabled by microelectromechanical systems (MEMS) (Figure 6) [28]. An IMU sensor with 9 axes that integrates accelerometers, gyroscopes, and magnetometers is commonly used for 6 DOF object tracking. Accelerometers measure the target’s acceleration. The angular velocity of a target is measured by a gyroscope. Additionally, a magnetometer detects the magnetic field strength at the target’s location. With sensor fusion of the raw measurements, an IMU sensor is able to obtain more accurate readings [29]. As a result, after calibration and compensation for drifts and errors, the position and orientation of the target can be determined [27].

The tri-axial measurements of the accelerometer are accelerations of each axis, where $a={\left[a_{x},a_{y},a_{z}\right]}^{T}={\left[\frac{d^{2}x}{dt^{2}},\frac{d^{2}y}{dt^{2}},\frac{d^{2}z}{dt^{2}}\right]}^{T}$. Readings from the gyroscope indicate the angular rates of the sensor when rotated, where $\omega ={\left[\omega_{x},\omega_{y},\omega_{z}\right]}^{T}={\left[\frac{d^{2}\varphi }{dt^{2}},\frac{d^{2}\theta }{dt^{2}},\frac{d^{2}\psi }{dt^{2}}\right]}^{T}$. Additionally, ${\left[pitch,roll,yaw\right]}^{T}$ is denoted as $\Phi ={\left[\varphi ,\theta ,\psi \right]}^{T}$.

By taking integration of the angular velocity from time $t_{k-1}$ to $t_{k}$,

(26) $$\Phi =\overset{t_{k}}{\underset{t_{k-1}}{\int }}m\omega \left(\tau \right)\;d$$

where τ is the discrete time. The solution of the orientation, under the assumption, can be written as

(27) $$\Phi =\frac{\omega_{k-1}+\omega_{k}}{2}\left(t_{k}-t_{k-1}\right)$$

For simplicity, three rotation matrices were defined as follows.

$$R_{pitch}=\left[\begin{array}{ccc} cos\theta & 0 & -sin\theta \\ 0 & 1 & 0\\ sin\theta & 0 & cos\theta \end{array}\right]$$

$$R_{roll}=\left[\begin{array}{ccc} 1 & 0 & 0\\ 0 & cos\psi & sin\psi \\ 0 & -sin\psi & cos\psi \end{array}\right]$$

$$R_{yaw}=\left[\begin{array}{ccc} cos\varphi & -sin\varphi & 0\\ sin\varphi & cos\varphi & 0\\ 0 & 0 & 1 \end{array}\right]$$

The rotational matrix R is expressed as

(28) $$R=R_{pitch}\cdot R_{roll}\cdot R_{yaw}$$

Due to the effect of the Earth’s gravity,

(29) $$\dot{\upsilon }=Ra-g$$

where υ is the velocity of the object and $g={\left[0,0,9.8\right]}^{T}$.

According to the midpoint method, the velocity

(30) $$\upsilon_{k}=\upsilon_{k-1}+\left(\frac{R_{k-1}a_{k-1}+R_{k}a_{k}}{2}-g\right)\left(t_{k}-t_{k-1}\right)$$

Thus, the position $p={\left[x,y,z\right]}^{T}$ at time k is

(31) $$p_{k}=p_{k-1}+\upsilon_{k-1}\left(t_{k}-t_{k-1}\right)+\frac{1}{2}\left(\frac{R_{k-1}a_{k-1}+R_{k}a_{k}}{2}-g\right){\left(t_{k}-t_{k-1}\right)}^{2}$$

## 3. Tracking Systems

Different types of tracking systems have been developed and marketed over the past decade. In this section, the main tracking systems in the market are reviewed in terms of their technical specifications.

### 3.1. Optical Tracking Systems

A large number of manufacturers have developed various kinds of optical tracking systems for biomedical applications, as summarized in Table 1. The main technical specifications that relate to the tracking performances are measurement volume (or FOV), resolution, volumetric accuracy, average latency, and measurement rates. Due to angle of view, the shape of measurement volume is usually a pyramid, which can be represented by radius × width × height. Some manufacturers prefer to use the term “field of view”, i.e., horizontal degree × vertical degree, to show the dimension of work volume. In addition, the advances of cameras promote the resolution of the image, and further increase the volumetric accuracy. To date, some advanced optical tracking system can obtain 26 megapixels (MP) resolution with 0.03 mm volumetric accuracy [30]. However, the high resolution of the captured images will burden the processor for data analysis, causing the increase of average latency and reduce of measurement rates.

### 3.2. Electromagnetic Tracking Systems

Compared to optical tracking systems, electromagnetic tracking system can cover a larger volume of measurement space, but normally has lower position accuracy. Table 2 summarizes the specification of some representative, commercially available electromagnetic tracking systems. Since it does not require the transmission of light, electromagnetic tracking systems are promising in intracorporeal biomedical applications. For example, Polhemus Inc. (Colchester, VT, USA) developed a miniatured electromagnetic motion tracking sensors with outer diameter of 1.8 mm. It can be inserted into human vessel with a catheter for both position and orientation tracking [69].

### 3.3. Mechanical Tracking Systems

Unlike other tracking systems, the development of mechanical tracking systems, especially for biomedical applications, is limited. This might be due to the fact that mechanical tracking systems are usually bulky and heavy. Meta motion. Inc. presented a mechanical tracking system, named Gypsy 7, decades ago, which had a position accuracy of 0.125° [79]. However, this kind of exoskeleton system consists of 14 joint sensors and the total weight is 4 kg.

### 3.4. Acoustic Tracking Systems

Most commercially available acoustic tracking system is related to marine positioning, the use for in-door positioning is still in its infancy. Sonitor Technologies, Inc. developed a Forkbeard system, which applied 40 kHz US for echo location [80]. Although it can cover a floor, the volumetric accuracy is 1–2 feet, while the latency is 1–2 s. The nature of low accuracy and high latency of acoustic tracking hamper its applications in biomedical field. However, considering that US has the benefit of non-ionizing radiation, it might be promising for some specific biomedical applications.

### 3.5. Inertial Tracking Systems

Inertial tracking systems are also commercially available for many years. Some products that can be purchased on the market are summarized in Table 3. Due to the fact that it does not require both transmitters and receivers as optical tracking, electromagnetic tracking or acoustic tracking, the size of the inertial tracking device can be very compact, such as a dot [81]. This feature contributes to a friendly and comfortable condition for tracking objects, which hardly affect the normal motion of objects. However, the inertial tracking system can only provide a position information relatively, and it always needs the assistance from other kinds of tracking systems.

## 4. Biomedical Ultrasound Imaging Applications

Over the past decade, a range of commercial and research tracking systems have been developed for biomedical US imaging-related applications. Following sections categorically review the applications of different tracking systems reported till date.

### 4.1. Freehand 3D Ultrasound Imaging

Over the past few decades, US imaging has become a valuable tool in clinical diagnostic and therapeutic procedures across a broad range of fields, ranging from routine screening, early cancer detection, diagnosis of cardiovascular disease to real-time monitoring [105]. Compared with CT, MRI, and PET, US demonstrates the advantages of safe for patients (no risk of ionizing radiation or high magnetic fields), real-time imaging, portability, and low-cost [11,106,107]. In clinical practice, a handheld US probe typically composed of a 1D linear US transducer array is routinely used to generate 2D US images in real-time, displaying cross-sectional images of the human anatomy. While 2D US imaging offers several advantages for medical applications, it can only acquire selectively-sampled, cross-sectional slice images of a 3D anatomic structure, and the orientation of each image plane depends on how the operator positions the handheld probe (i.e., operator dependency) [108]. If clinicians need to view 3D anatomic structures, they have to imagine the 3D volume with the planar 2D images mentally, thus limiting the diagnostic accuracy.

In order to overcome the limitations of 2D US, volumetric 3D US imaging has been developed, allowing direct visualization of the arbitrary plane of 3D volume and helping obtain a more accurate view of the shape, size, and location of the organ and lesion [16]. Up to now, three different types of methods have been utilized for the construction of 3D US volumes: employing a 2D phased array transducer, mechanical 3D US scanning, and freehand 3D US scanning [109,110]. Instead of using 1D array ultrasonic transducer for conventional 2D US systems, 3D US volume can also be generated by using a 2D phased array ultrasonic transducer with its elements spreading on a 2D aperture, which can deflect and focus the ultrasonic beam in a volumetric space [111,112]. Since the US beams are steered and focused on the region of interest by electronic scanning, the 2D array remains stationary during the procedure. Although this approach can acquire 3D volume straightforwardly and in real-time, manufacturing process of a 2D phased array transducer is complex and manufacturing cost is high due to a large number of array elements and the electrical connection of each element [113]. Another approach to obtain a 3D US volume is via mechanical 3D scanning using conventional linear array transducer. In this method, a mechanical motor is used to control the transducer rotation, tilt, or translation with designed scanning trajectory [109]. The 3D US volumes can then be reconstructed by using the acquired 2D US images with their predefined positions and orientations. While the 3D US imaging systems based on this kind of method can be operated conveniently by controlling mechanical motor, the whole system is bulky due to a mechanical motor integrated and the system flexibility is low due to the controlled movement limitation.

**Figure 7:**
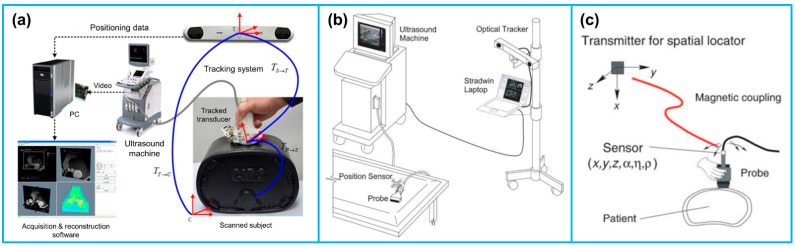
(**a**) The typical configuration of a freehand 3D US imaging system. Reprinted from [115] with permission. (**b**) An optical tracker based freehand 3D US imaging system. Reprinted from [109] with permission. (**c**) An electromagnetic sensor based freehand 3D US imaging system. Reprinted from [109] with permission.

In addition to the above-mentioned approaches for 3D US imaging, freehand 3D US has become the most rapidly advancing technique over the years due to the advantages of scanning flexibility, convenience to operate and low cost. Freehand 3D US images are acquired by rigidly attaching a 6-DOF position sensor to a handheld US probe that generates a sequence of B-mode US images [114]. The position sensor records the positions and orientations of the probe during the scanning procedure, and then the 3D volumes are constructed by combining the sequence of the 2D US images along with the corresponding position information (Figure 7a). It is noted that for reconstructing a 3D US volume, the position and orientation data of each 2D US image is required. Various techniques have been reported for obtaining the position and orientation data of the US probe during freehand US scanning. The most commonly used position sensors during freehand 3D US imaging are optical tracking sensor and electromagnetic tracking sensor.

In a typical optical tracking system, either light-reflective makers (passive markers) or light-emitting markers (active markers) are attached to the US probe and the markers are monitored by two or more cameras fixed in a position (Figure 7b). Passive markers are usually matt spheres coated with retroreflective material and reflect light back to the cameras. Three or more markers are usually arranged asymmetrically, allowing the cameras to infer the orientation in space. Contrary to passive markers that reflect light generated by the external sources to the cameras, active markers are made of infrared LEDs, powered by themselves to emit infrared light. In a typical electromagnetic tracking system, a time-varying 3D magnetic field is transmitted through the volume in which the US scanning is to be conducted. Three sensor coils are attached to an US probe and utilized to obtain the field in the 3D Cartesian coordinates (x, y, z) (Figure 7c). This information enables the position and orientation of the sensor coils to be acquired [109].

**Figure 8:**
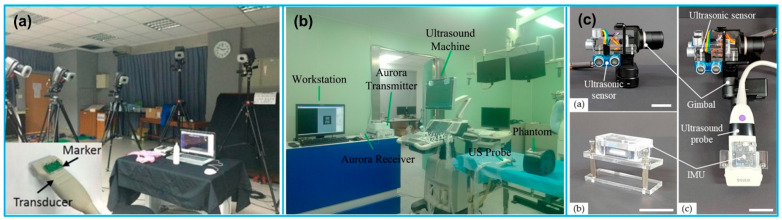
(**a**) A freehand 3D US imaging system with optical motion tracking system settings. Reprinted from [116] with permission. (**b**) The setup of a freehand 3D US imaging system with electromagnetic tracking. Reprinted from [122] with permission. (**c**) A low-cost 3D US image acquisition method. Reprinted from [126] with permission.

Due to the advantages of flexible operation and simultaneous visualization, freehand 3D US imaging is increasingly gaining popularity in medical applications. For example, Chung et al. [116] reported an imaging system based on optical motion tracking technique with the objective of developing a carotid artery contour detection procedure for carotid atherosclerosis diagnosis (Figure 8a). The 3D motion tracking system consisted of 8 Eagle digital CCD cameras for motion detection in 3D space and 4 passive fluorescent markers attached to an US probe, showing spatial and temporal resolutions of 10 μm and 0.01 s, respectively. Daoud et al. [117] developed a freehand 3D US imaging system using a 3D electromagnetic position tracking system (trakSTAR, NDI, ON, Canada). The position and orientation of the US probe in 3D space were tracked by one of the electromagnetic sensors attached to the probe (Figure 8b). Herickhoff et al. [17] invented a volumetric 3D US imaging system at a very low cost (under USD 250) by using a single IMU sensor for orientation acquisition and a light-weight fixture customized to the US probe (Figure 8c). The preliminary results demonstrated the capability of the low-cost method for reconstructing a 3D US image volume, providing a solution for solving the problem of operator dependence. In another study, Chen and Huang [118] reported a freehand 3D US imaging system that could obtain volume reconstruction and visualization during data acquisition at real-time level. The real-time freehand 3D US system mainly consisted of a linear probe, an electromagnetic sensing system, and a computer with a GPU for image data reconstruction and visualization of the 3D volume image. A summary of the various reported freehand 3D US imaging system during the past decade is provided in Table 4.

**Table 4:** A summary of freehand 3D US imaging study.

Reference	Tracking Principle	Tracking System	Accuracy	Application
Chung et al. [116]	Optical tracking	Motion Analysis, Santa Rosa, CA, USA	Spatial: 10 μm Temporal: 0.01 s	Carotid atherosclerotic stenosis detection
Pelz et al. [119]	Electromagnetic tracking	Curefab CS system (Curefab Technologies GmbH, Munich, Germany)	None	Internal carotid artery stenosis diagnosis
Miller et al. [120]	Optical tracking	VectorVision2 navigation system (BrainLAB, Munich, Germany)	None	Image-guided surgery
Mercier et al. [121]	Optical tracking	Polaris (Northern Digital, Waterloo, ON, Canada)	Spatial: 0.49–0.74 mm Temporal: 82 ms	Neuronavigation
Chen et al. [122]	Electromagnetic tracking	Aurora (NDI, ON, Canada)	None	Image-guided surgery
Wen et al. [115]	Optical tracking	Polaris (Northern Digital, Waterloo, ON, Canada)	None	Image-guided intervention
Sun et al. [123]	Optical tracking	OptiTrack V120:Trio (NaturalPoint Inc., Corvallis, OR, USA)	Spatial: <1 mm	Image-guided intervention
Worobey et al. [124]	Optical tracking	Vicon Motion Systems; Centennial, Colorado	None	Scapular position
Passmore et al. [125]	Optical tracking	Vicon Motion Systems, Oxford, UK	None	Femoral torsion measurement
Daoud et al. [117]	Electromagnetic tracking	trakSTAR, NDI, ON, Canada	None	3D US imaging
Chen and Huang [118]	Electromagnetic tracking	MiniBird, Ascension Technology Corp., Burlington, VT, USA	None	Real-time 3D imaging
Cai et al. [20]	Optical tracking	OptiTrack V120: Trio (NaturalPoint Inc., Corvallis, OR, USA)	Positional: 0.08–0.69 mm Rotational: 0.33–0.62°	3D US imaging
Herickhoff et al. [17]	Inertial tracking	IMU sensor (iNEMO-M1; STMicroelectronics, Geneva, Switzerland)	None	Low-cost 3D imaging platform
Kim et al. [126]	Inertial tracking	Ultrasonic sensor + IMU sensor (HC-SR04, Shenzhen AV, Shenzhen, China)	Spatial: 0.79–1.25 mm	Low-cost 3D imaging platform
Lai et al. [127]	Optical tracking	T265, Intel, Santa Clara, CA, USA	Spatial: 2.9 ± 1.8°	Scoliosis assessment
Jiang et al. [128]	Electromagnetic tracking	Ascension Technology, Burlington, VT, USA	None	Scoliosis assessment

### 4.2. Ultrasound Image Fusion in Multimodality Imaging

**Figure 9:**
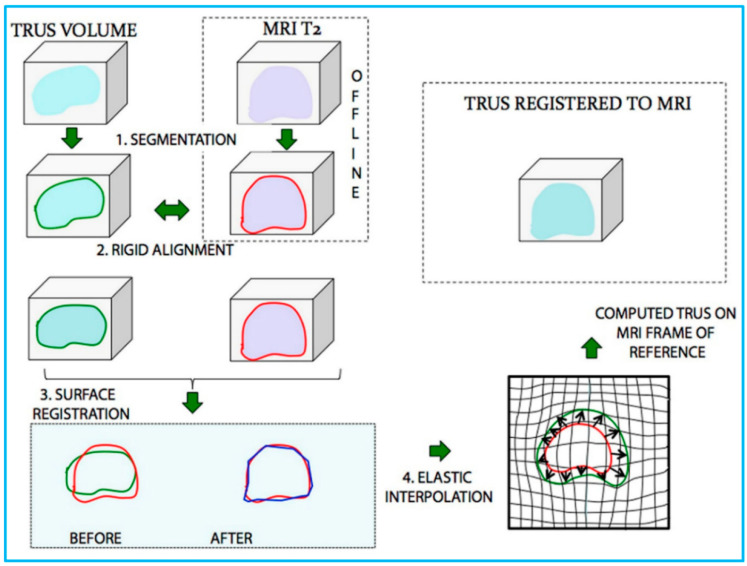
Schematic illustration of the process of US/MRI fusion. MR and transrectal US (TRUS) images were (1) segmented and then (2) rigidly aligned. Fusion then proceeded, involving (3) a surface registration, and (4) elastic (non-rigid) interpolation. Reprinted from [135] with permission.

Medical image fusion refers to the co-display of registered images from the same or different imaging modalities, such as US, CT, MRI, and PET [129]. Since the fused image contains all the important features from each input image, it can offer a more comprehensive, more reliable and better description of lesions, so as to assist the preclinical research and clinical diagnosis as well as therapy, such as routine staging, surgical navigation, radiotherapy planning, etc. [130] Percutaneous interventional procedures, particularly percutaneous biopsy and percutaneous tumor ablation, play an important role in caring for patients with cancer. To guide percutaneous interventional procedures, US imaging is the most widely used imaging modality owing to its real-time capability, no radiation exposure, and easy accessibility [131]. However, compared with CT and MRI, US imaging shows a narrower field of view and lower contrast resolution. In addition, the imaging performance is reduced by the presence of gas and fat in human body [132]. To localize and characterize lesions more precisely, applying US fusion imaging allows exploitation of the strengths of different imaging modalities simultaneously, eliminating or minimizing the weakness of every single modality [133]. The procedures of fusing CT/MRI images and US images are detailed in reference [134], the interested readers can refer to it. After the image fusion procedure, the CT/MRI images will be displayed on the monitor side-by-side with the real-time US images in a synchronous manner and updated simultaneously according to the change in position and imaging plane of US probe. A process of US and MRI fusion is illustrated in Figure 9.

**Figure 10:**
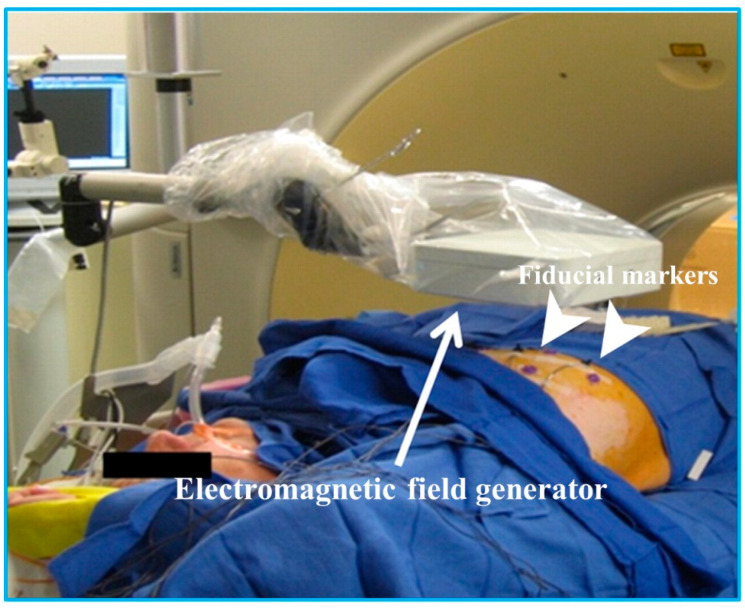
Equipment setup for electromagnetic tracking during interventional procedures. Reprinted from [138] with permission.

As we have discussed in Section 3, to track an US probe in 3D space, there are 5 available tracking techniques. However, for US image fusion applications in percutaneous interventional procedures, the electromagnetic tracking system is the one mostly implemented [129], as shown in Figure 10. For instance, Krucker et al. [136] developed an Aurora (Northern Digital Inc, Waterloo, ON, Canada) electromagnetic tracking system to fuse real-time US with CT, providing real-time visualization of tracked interventional needles within preprocedural CT scans. Appelbaum et al. [137] compared conventional CT-guided biopsy to biopsy employing a U.S. Food and Drug Administration–approved electromagnetic biopsy navigation system (Veran IG4, Veran Medical Technologies). Phantom model study results showed that by using electromagnetic tracking system, needle placement accuracy had been improved and radiation exposure had been reduced compared with conventional CT techniques. Venkatesan et al. [138] fused US image to CT and ^18^F-FDG-PET/CT with an electromagnetic tracking system (Northern Digital Inc, Waterloo, ON, Canada) for biopsy of technically challenging FDG-avid targets. By using conventional US imaging, a total number of 36 lesion samples could not be well seen or were completely inapparent during the biopsy procedures. However, by using the combined electromagnetic tracking and US/CT/^18^F-FDG-PET fusion, 31 out of 36 biopsies were diagnostic.

**Figure 11:**
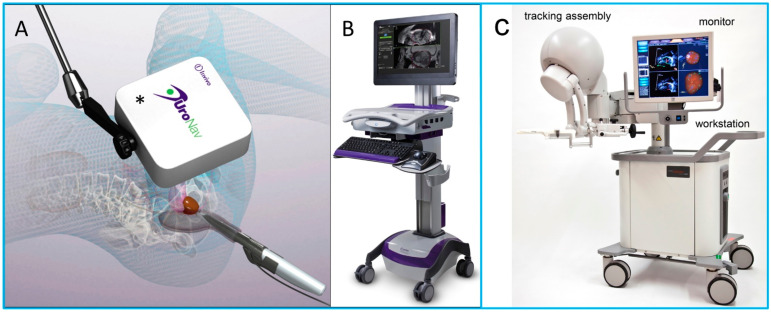
(**A**,**B**) Photo images of the UroNav US/MRI fusion system. (**A**) An electromagnetic field generator enables tracking of the transrectal US probe; (**B**) US/MRI fusion device. (**C**) Artemis US/MRI fusion system. Reprinted from [144] with permission.

In recent decade, US image fusion has developed significantly and can now perform crucial roles in diagnosis and clinical management across various anatomical regions [129,139,140,141,142,143]. One of the most widely applied examples in clinics is US fused with MRI images for percutaneous image-guided prostate biopsy [144,145]. Although US is the commonest modality utilized for real-time guidance during biopsy, it is limited in its ability to visualize deep targets. In addition, the biopsy procedure is performed targeting only the different anatomic locations of the prostate, thus the underdetection rate of transrectal US-guided biopsy is high. Fusing US images with MRI images allows the information from MRI to be used to direct biopsy needles under US guidance. It combines the superior diagnostic accuracy of MRI for detecting suspicious lesions in the prostate with the practicality and familiarity of US [145,146]. Several U.S. FDA approved systems for fusion imaging of real-time US with MRI are commercially available (summarized in Table 5), as shown in Figure 11.

In addition to US/MRI fusion-guided prostate biopsy, US image fusion has been investigated for clinical applications in various anatomical regions including liver, kidney, pancreas, and musculoskeletal system. A summary of US image fusion for applications in different anatomical regions is illustrated in Table 6.

### 4.3. Ultrasound-Guided Diagnosis

Percutaneous needle biopsy plays an important role in the diagnosis, staging, and treatment planning for various tumors [155,156]. The success of needle insertion procedures mainly depends on accurate needle placement to minimize complications and to avoid damage to neighboring tissues [156]. In many applications, US guidance has been shown to increase the safety and success rate of the procedure due to its real-time imaging capability, easy operation, portability, etc. [157,158,159]. During the procedure, the physician manually manipulates the needle and the US probe simultaneously while mentally relating US images acquired to locations inside a patient’s body [160]. Practically, it is very challenging for the physician to visualize the needle trajectory inside the patient tissue just by checking the US image [156]. In order to let the needle tip follow the desired trajectory and hit the target location in the image plane, it is beneficial and necessary to track the pose of the needle with respect to the coordinate system of the US image.

**Figure 12:**
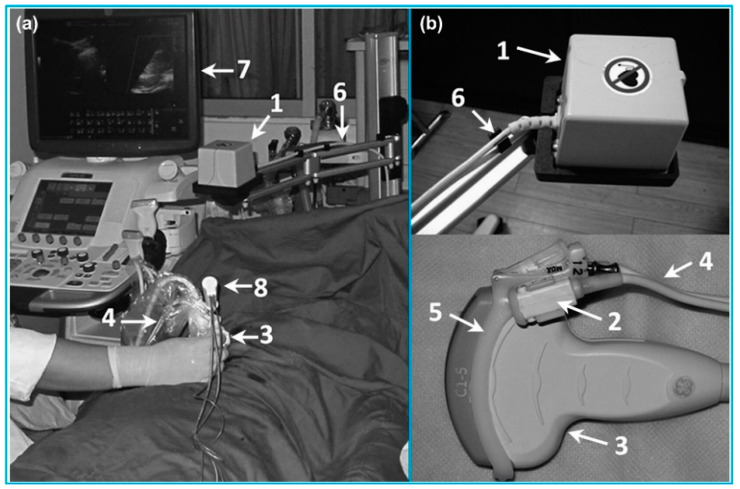
A magnetic navigation system for liver cancer ablation procedures. (**a**) The setup of the magnetic navigation system; (**b**) Magnetic field generator and magnetic receivers attached to an US probe. Reprinted from [162] with permission.

Three different types of tracking systems have been applied for US-guided needle insertion: electromagnetic, optical and mechanical trackers. For electromagnetic trackers, Franz et al. [161] assessed the precision and accuracy of a compact electromagnetic field generator (Aurora, Northern Digital Inc., Waterloo, ON, Canada) attached to 6 different US probes with various operating frequencies. Based on the assessment results, the error of the field generator was <0.2 mm; the positional accuracy was <1.0 mm. Xu et al. [162] evaluated the effectiveness of magnetic navigation in US-guided interventional procedures (Figure 12). A commercially available magnetic navigation system (GE Healthcare, Milwaukee, WI, USA) was applied. They found that compared with conventional US guidance, magnetic navigation in US-guided interventional procedure was especially useful for some complicated clinical situations, such as liver tumor ablation. In addition, Hakime et al. [163] evaluated the accuracy and safety of electromagnetic needle tracking for US-guided liver biopsy. An electromagnetic transmitter was placed near the scanning area and a pair of electromagnetic receiving sensors were attached to the US probe. The clinical results demonstrated that the overall diagnostic success rate of liver lesion was 91%. März et al. [164] proposed an interventional imaging system based on a mobile electromagnetic field generator (Aurora, Northern Digital Inc., Waterloo, ON, Canada) attached to an US probe. The tracking and calibration accuracy of the system was assessed in a clinical setting. The tracking accuracy was tested to be <1 mm and the calibration error was 1–2 mm.

**Figure 13:**
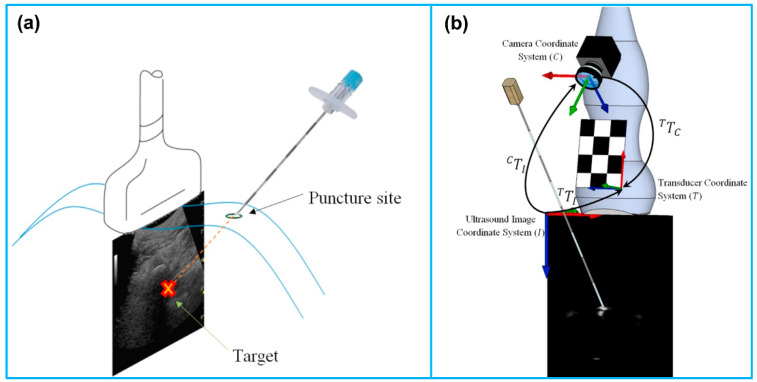
(**a**) Out-of-plane needle trajectory planning. (**b**) Schematic of the coordinate systems and needle movement. Reprinted from [167] with permission.

For optical trackers, Wang et al. [165] utilized a low-cost Kinect sensor (a stereo camera) for interventional needle tracking. The accuracy of needle tracking was measured, ranging from 2.6 ± 1.7 to 6.9 ± 5.1 mm. Stolka et al. [166] developed a camera-based tracking system for US-guided interventions, consisting of an optical sensing head mounted on an US probe. The head could be mounted to support both in- or out-of-plane interventions. The phantom test results showed that the mean accuracy of the system was 3.27 ± 2.28 mm. Najafi et al. [167] proposed a single camera-based tracking system for US-guided needle insertion (Figure 13). The camera was directly mounted on the US probe and the needle location was tracked by using the needle markers. A needle tracking accuracy of 0.94 ± 0.46 mm was achieved, which was higher than that of the existing solutions. Daoud et al. [168] also reported a camera-based tracking system for US-guided needle interventions. An USB web camera (IceCam2, Macally Peripherals, Ontario, CA, USA) was attached to a 3D curvilinear US probe using a plastic housing. Dynamic needle tracking in a sequence of 3D US volumes was achieved. Based on the ex vivo animal experiments, the maximum error rate of 1.2 mm for the needle tip was measured in individual US volumes.

**Figure 14:**
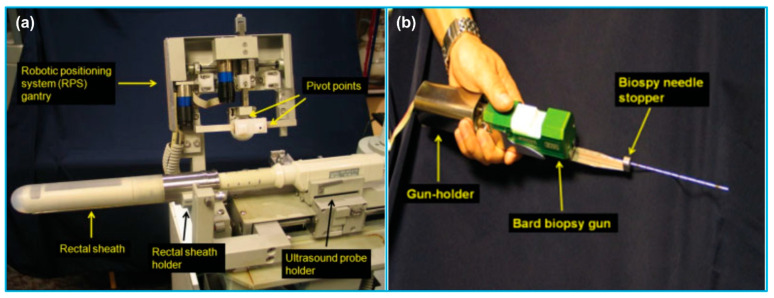
A robotic US-guided prostate intervention system. (**a**) Gantry and US probe holder. (**b**) Gun-holder and biopsy gun. Reprinted from [169] with permission.

In addition to the magnetic and optical tracking devices, Ho et al. [169] invented an US-guided robotic system for transperineal prostate intervention, consisting of a gantry, a gun-holder, and an US probe holder (Figure 14). The system was constructed based on the dual-cone concept, ensuring that any part of the prostate can be accessed with minimal skin puncture. The egg phantom experimental results illustrated the system accuracy was <1 mm. Orhan et al. [170] reported design and modeling of a 5-DOF parallel robot for autonomous US-guided biopsy. The robot was composed of 5-DOF and 3 main stages; front stage, back stage, syringe mechanism. The biopsy needle connected to the syringe mechanism passed through the gimbal in the front stage. Poquet et al. [171] designed a 6-DOF, serial robotic co-manipulator system for assisting endorectal prostate biopsy. The robotic system consisted of three brakes and three motors. The system could provide freedom to the urologist to position the probe with respect to the prostate in the free mode while leaving him/her to focus on insertion only during locked mode.

### 4.4. Ultrasound-Guided Therapy

**Figure 15:**
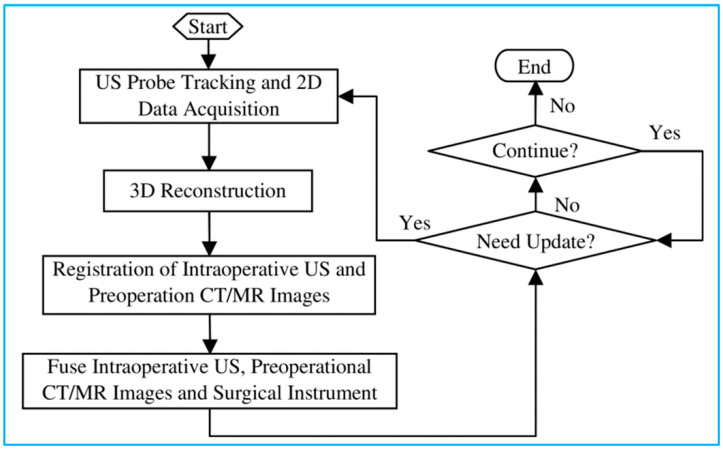
A basic process of 3D US image-guided surgery navigation. Reprinted from [172] with permission.

US-guided surgery is an area of minimally invasive surgery where surgical procedures are performed with the aid of US imaging throughout the operation. Contrary to traditional surgical access, US-guided surgery uses computer-based systems to provide real-time US images to help the physician precisely visualize and target the surgical site by updating the intraoperative information [12]. While other imaging modalities, such as CT and MRI, have also been applied for surgery navigation, US-guided surgery shows several advantages, including real-time imaging, equipment portability, low cost and reduced hospital stays [13]. Figure 15 shows a basic process of 3D US-guided surgery navigation [172]. In order to utilize US to guide surgical procedures, the US probe must be tracked. Although several tracking technologies are commercially available today, which are review in the last section, the most widely used solutions are optical and electromagnetic systems.

**Figure 16:**
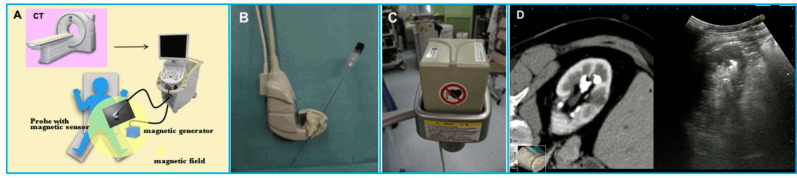
A navigation system for percutaneous renal puncture. (**A**) The main components of the system; (**B**) The magnetic sensor attached to an US probe; (**C**) The magnetic field generator; (**D**) US image and CT volume data displaying side by side on the same monitor. Reprinted from [175] with permission.

For instance, Stoll et al. [173] presented a novel approach for tracking surgical instruments in 3D US imaging by using a series of passive echogenic markers. The markers were attached near the distal end of the surgical instrument, and the marker position and orientation could be simply determined in a 3D US volume using image processing. Since the markers were completely passive, they can be easily implemented without prior integration with the imaging system. Moreover, the error of registering the tracking coordinate frame to the image frame can be eliminated. In another study, Li et al. [174] systematically compared the real-time US-guided percutaneous nephrolithotomy (PCNL) using SonixGPS navigation system with conventional US-guided PCNL using an US machine for the treatment of complex kidney stones. Based on their clinical results, the SonixGPS system was superior to the conventional method in terms of stone clearance rate and puncture accuracy. Hamamoto et al. [175] investigated the efficacy of applying real-time virtual sonography (RVS) guidance for renal puncture for endoscopic combined intrarenal surgery (ECIRS) treatment of large renal calculi (Figure 16). The RVS system synchronized real-time US images with CT images via a magnetic navigation system to provide volume and position data side by side. Compared with US-guided puncture, RVS-guided renal puncture illustrated lower incidence of bleeding-related complications. In addition, Gomes-Fonseca et al. [176] assessed the performance of electromagnetic tracking system guidance for percutaneous renal access in the operating room environment. Their experimental results demonstrated that ureterorenoscopes and 2D US probe did not affect the precision and accuracy of the electromagnetic tracking systems, suggesting that these instruments may be used for a safe percutaneous renal access.

**Figure 17:**
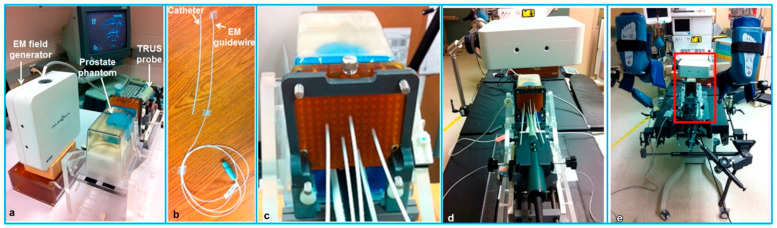
The experimental setup for catheter tracking in a controlled laboratory environment (**a**–**c**) and in a brachytherapy operating room (**d**,**e**). (**a**) The experimental phantom setup; (**b**) The flexible electromagnetic-tracked guidewire and catheter; (**c**) Catheters inserted into the prostate model through the grid; (**d**) The experimental setup positioned on the treatment table in the operating room; (**e**) Mimicking a typical brachytherapy setup. Reprinted from [177] with permission.

Bharat et al. [177] measured the accuracy of the electromagnetic tracking system for identification of the position and shape of the treatment catheters in high-dose-rate (HDR) prostate brachytherapy (Figure 17). The tracking experiments were performed in both a controlled laboratory environment and a typical brachytherapy operating room. The robotic validation of the electromagnetic system found that the mean accuracy of the system was <0.5 mm, illustrating the potential value of using electromagnetic tracking for catheter mapping in HDR brachytherapy. Schwaab et al. [178] developed an US based motion tracking method for real-time motion correction in ion beam therapy. It was found that by using US tracking, it can yield nearly real-time position information at high frame rate of moving targets. Yu et al. [179] also evaluated the accuracy and precision of a transperineal US image-guided system (Clarity Autoscan US system (Elekta, Stockholm, Sweden)) for prostate radiotherapy. Based on a male pelvic phantom experimental result, the accuracy of US tracking performance in the lateral direction was better than that in the axial direction; the precision of US tracking performance in the axial (superior-inferior) direction was better than that in the lateral (left-right) direction.

**Figure 18:**
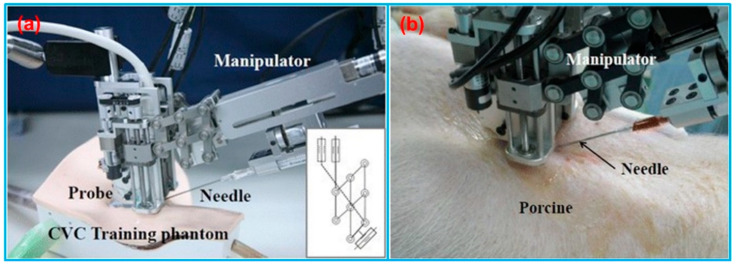
(**a**) Needle insertion manipulator for central venous catheterization. (**b**) Overview of venous puncture experiment in a porcine model. Reprinted from [182] with permission.

In addition to US-guided surgical navigation and radiotherapy, US-guided catheterization has also attracted the attention of many researchers. Jakola et al. [180] reported a method to guide the placement of ventricular catheters using 3D US navigation system. The US-based navigation system (Sonowand Invite, Sonowand AS, Trondheim, Norway) consisted of an US probe integrated with an optical tracking system. Based on the patient studies, this 3D US navigation system was promising for accurate placement of catheters. Brattain et al. [181] designed a probe-mounted US guidance system for US-guided procedures. The system consisted of a lockable, articulating needle guide that attached to an US probe and a user-interface that provided real-time visualization of the predicted needle trajectory overlaid on the US image. The system illustrated the potential to increase efficiency, safety, quality, and reduce costs for US-guided procedures. Kobayashi et al. [182] invented an US-guided needle insertion manipulator for central venous catheterization (Figure 18). The performance of the manipulator was evaluated in vivo in a porcine model. The animal study results found that a venous placement rate of 80% could be obtained with opened skin, and this system was especially effective for jugular venous puncture of opened skin.

## 5. Conclusions

In this paper, we categorized and reviewed different types of tracking devices for biomedical US imaging applications based on the different tracking principles. The applications of various tracking systems reported in the literature in the past decade were categorized into four types: freehand 3D US imaging, US image fusion, US-guided diagnosis as well as US-guided therapy. In this review article, the working principles of different tracking technologies were analyzed in terms of their advantages and disadvantages for biomedical applications. A comprehensive overview of the state-of-the-art tracking devices on the market is provided in terms of their technical specifications, including accuracy, update rate and latency. With the rapid advancement of various tracking devices over the past decade, the usefulness of different tracking systems has been illustrated by a diverse range of biomedical applications, as reviewed in this paper.

## 6. Future Perspectives

Although the utilization of tracking device is becoming more and more essential for providing better information and navigation for biomedical applications, there is still much room for improvement. Nowadays, many different types of commercial tracking devices have been introduced and no significant specification differences have been found among them. For the biomedical applications, such as image-guided surgery, perhaps the existing tracking technologies do not fully meet the requirements, and the best choice of tracking device is highly application dependent. The future research of tracking systems may be focused on further improving accuracy and reducing the registration error of these technologies for medical applications. While freehand 3D US has already demonstrated its benefits for obstetrics, cardiology, and image-guided intervention applications, more preclinical studies are required to allow physicians to integrate 3D US imaging effectively and safely into US-guided interventional procedures. In addition, while real-time US image fusion has demonstrated its usefulness in different anatomical regions, such as prostate, liver, and kidney, future studies need to explore its effectiveness in imaging other anatomical regions or during surgery. Although the advancement of different tracking devices has accelerated the development of US image-guided systems, most of these systems are still in the prototype stage, and so far, only limited clinical trials have been carried out. As surgery continues to move toward minimally invasive interventions, US image-guided systems will increasingly be used to improve the precision and quality of medical procedures. More studies from the fields of biomedical engineering, medical physics as well as clinical research are necessary to move this technology from laboratory to hospital to improve patient care.

## Figures and Tables

**Table 1:** Summary of commercially available optical tracking systems.

Manufacturer	Model	Measurement Volume (Radius × Width × Height) or FOV	Resolution	Volumetric Accuracy (RMS)	Average Latency	Measurement Rate
Northern Digital Inc., Waterloo, ON, Canada	Polaris Vega ST [31]	2400 × 1566 × 1312 mm^3^ (Pyramid Volume:) 3000 × 1856 × 1470 mm^3^ (Extended Pyramid)	N/A	0.12 mm (Pyramid Volume) 0.15 mm (Extended Pyramid)	<16 ms	60 Hz
	Polaris Vega VT [32]	2400 × 1566 × 1312 mm^3^ (Pyramid Volume) 3000 × 1856 × 1470 mm^3^ (Extended Pyramid)	N/A	0.12 mm (Pyramid Volume) 0.15 mm (Extended Pyramid)	<16 ms	60 Hz
	Polaris Vega XT [33]	2400 × 1566 × 1312 mm^3^ (Pyramid Volume) 3000 × 1856 × 1470 mm^3^ (Extended Pyramid)	N/A	0.12 mm (Pyramid Volume) 0.15 mm (Extended Pyramid)	<3 ms	400 Hz
	Polaris Vicra [34]	1336 × 938 × 887 mm^3^	N/A	0.25 mm	N/A	20 Hz
ClaroNav Inc., Toronto, ON, Canada	H3-60 [35]	2400 × 2000 × 1600 mm^3^	1280 × 960	0.20 mm	~60 ms	16 Hz
	SX60 [35]	1150 × 700 × 550 mm^3^	640 × 480	0.25 mm	~20 ms	48 Hz
	HX40 [35]	1200 × 1200 × 900 mm^3^	1024 × 768	0.20 mm	~50 ms	20 Hz
	HX60 [35]	2000 × 1300 × 1000 mm^3^	1024 × 768	0.35 mm	~50 ms	20 Hz
BTS Bioengineering Corp., Quincy, MA, USA	SMART DX 100 [36]	2000 × 2000 × 2000 mm^3^	0.3 MP	<0.20 mm	N/A	280 FPS
	SMART DX 400 [36]	4000 × 3000 × 3000 mm^3^	1.0 MP	<0.30 mm	N/A	300 FPS
	SMART DX 700 [36]	4000 × 3000 × 3000 mm^3^	1.5 MP	<0.10 mm	N/A	1000 FPS
	SMART DX 6000 [36]	4000 × 3000 × 3000 mm^3^	2.2 MP	<0.10 mm	N/A	2000 FPS
	SMART DX 7000 [36]	6000 × 3000 × 3000 mm^3^	4.0 MP	<0.10 mm	N/A	2000 FPS
NaturalPoint, Inc., Corvallis, OR, USA	OptiTrack Prime^X^ 41 [37]	FOV 51° × 51°	4.1 MP	0.10 mm	5.5 ms	250+ FPS
	OptiTrack Prime^X^ 22 [38]	FOV 79° × 47°	2.2 MP	0.15 mm	2.8 ms	500+ FPS
	OptiTrack Prime^X^ 13 [39]	FOV 56° × 46°	1.3 MP	0.20 mm	4.2 ms	1000 FPS
	OptiTrack Prime^X^ 13W [40]	FOV 82° × 70°	1.3 MP	0.30 mm	4.2 ms	1000 FPS
	OptiTrack Slim^X^ 13 [41]	FOV 82° × 70°	1.3 MP	0.30 mm	4.2 ms	1000 FPS
	OptiTrack V120: Trio [42]	FOV 47° × 43°	640 × 480	N/A	8.33 ms	120 FPS
	OptiTrack V120: Duo [43]	FOV 47° × 43°	640 × 480	N/A	8.33 ms	120 FPS
	OptiTrack Flex 13 [44]	FOV 56° × 46°	1.3 MP	N/A	8.3 ms	120 FPS
	OptiTrack Flex 3 [45]	FOV 58° × 45°	640 × 480	N/A	10 ms	100 FPS
	OptiTrack Slim 3U [46]	FOV 58° × 45°	640 × 480	N/A	8.33 ms	120 FPS
	TrackIR 4 [47]	46° (Horizontal)	355 × 288	N/A	N/A	120 FPS
	TrackIR 5 [47]	51.7° (Horizontal)	640 × 480	N/A	N/A	120 FPS
Qualisys Inc., Gothenburg, Sweden	Arqus A5 [30]	FOV 77° × 62°	5 MP (normal) 1MP (high-speed)	0.06 mm	N/A	700 FPS (normal) 1400 FPS (high-speed)
	Arqus A9 [30]	FOV 82° × 48°	9 MP (normal) 2.5 MP (high-speed)	0.05 mm	N/A	300 FPS (normal) 590 FPS (high-speed)
	Arqus A12 [30]	FOV 70° × 56°	12 MP (normal) 3 MP (high-speed)	0.04 mm	N/A	300 FPS (normal) 1040 FPS (high-speed)
	Arqus A26 [30]	FOV 77° × 77°	26 MP (normal) 6.5 MP (high-speed)	0.03 mm	N/A	150 FPS (normal) 290 FPS (high-speed)
	Miqus M1 [48]	FOV 58° × 40°	1 MP	0.14 mm	N/A	250 FPS
	Miqus M3 [48]	FOV 80° × 53°	2 MP (normal) 0.5 MP (high-speed)	0.11 mm	N/A	340 FPS (normal) 650 FPS (high-speed)
	Miqus M5 [48]	FOV 49° × 49°	4 MP (normal) 1 MP (high-speed)	0.07 mm	N/A	180 FPS (normal) 360 FPS (high-speed)
	Miqus Hybrid [49]	FOV 62° × 37°	2 MP	N/A	N/A	340 FPS
	3+ [50]	N/A	1.3 MP (normal) 0.3 MP (high-speed)	N/A	N/A	500 FPS (normal) 1750 FPS (high-speed)
	5+ [50]	49° (Horizontal)	4 MP (normal) 1 MP (high-speed)	N/A	N/A	180 FPS (normal) 360 FPS (high-speed)
	6+ [50]	56° (Horizontal)	6 MP (normal) 1.5 MP (high-speed)	N/A	N/A	450 FPS (normal) 1660 FPS (high-speed)
	7+ [50]	54° (Horizontal)	12 MP (normal) 3 MP (high-speed)	N/A	N/A	300 FPS (normal) 1100 FPS (high-speed)
Vicon Industries Inc., Hauppauge, NY, USA	Valkyrie VK26 [51]	FOV 72° × 72°	26.2 MP	N/A	N/A	150 FPS
	Valkyrie VK16 [51]	FOV 72° × 56°	16.1 MP	N/A	N/A	300 FPS
	Valkyrie VK8 [51]	FOV 72° × 42°	8.0 MP	N/A	N/A	500 FPS
	Valkyrie VKX [51]	FOV 66° × 66°	7.2 MP	N/A	N/A	380 FPS
	Vantage+ V16 [52]	FOV 76.4° × 76.4°	16 MP (normal) 4.2 MP (high-speed)	N/A	8.3 ms	120 FPS (normal) 500 FPS (high-speed)
	Vantage+ V8 [52]	FOV 61.7° × 47°	8 MP (normal) 2.2 MP (high-speed)	N/A	5.5 ms	260 FPS (normal) 910 FPS (high-speed)
	Vantage+ V5 [52]	FOV 63.5° × 55.1°	5 MP (normal) 1.8 MP (high-speed)	N/A	4.7 ms	420 FPS (normal) 1070 FPS (high-speed)
	Vero v2.2 [53]	FOV 98.1° × 50.1°	2.2 MP	N/A	3.6 ms	330 FPS
	Vero v1.3 [53]	FOV 55.2° × 43.9°	1.3 MP	N/A	3.4 ms	250 FPS
	Vero v1.3 X [53]	FOV 79.0° × 67.6°	1.3 MP	N/A	3.4 ms	250 FPS
	Vero Vertex [53]	FOV 100.6° × 81.1°	1.3 MP	N/A	3.4 ms	120 FPS
	Vue [54]	FOV 82.7° × 52.7°	2.1 MP	N/A	N/A	60 FPS
	Viper [55]	FOV 81.8° × 49.4°	2.2 MP	N/A	3.2 ms	240 FPS
	ViperX [56]	FOV 50.2° × 50.2°	6.3 MP	N/A	3.2 ms	240 FPS
Atracsys LLC., Puidoux, Switzerland	fusionTrack 500 [57]	2000 × 1327 × 976 mm^3^	2.2 MP	0.09 mm	~ 4 ms	335 Hz
	fusionTrack 250 [58]	1400 × 1152 × 900 mm^3^	2.2 MP	0.09 mm	~ 4 ms	120 Hz
	spryTrack 180 [59]	1400 × 1189 × 1080 mm^3^	1.2 MP	0.19 mm	<25 ms	54 Hz
	spryTrack 300 [60]	1400 × 805 × 671 mm^3^	1.2 MP	0.14 mm	<25 ms	54 Hz
Motion Analysis Corp., Rohnert Park, CA, USA	Kestrel 4200 [61]	N/A	4.2 MP	N/A	N/A	200 FPS
	Kestrel 2200 [62]	N/A	2.2 MP	N/A	N/A	332 FPS
	Kestrel 1300 [63]	N/A	1.3 MP	N/A	N/A	204 FPS
	Kestrel 300 [64]	N/A	0.3 MP	N/A	N/A	810 FPS
STT Systems, Donostia-San Sebastian, Spain	EDDO Biomechanics [65]	N/A	N/A	1 mm	N/A	120 FPS
Advanced Realtime Tracking GmbH & Co. KG, Oberbayern, Germany	ARTTRACK6/M [66]	FOV 135° × 102°	1280 × 1024	N/A	N/A	180 Hz
	ARTTRACK5 [67]	FOV 98° × 77°	1280 × 1024	N/A	10 ms	150 Hz
	SMARTTRACK3 [68]	FOV 135° × 102°	1280 × 1024	N/A	9 ms	150 Hz

**Table 2:** Summary of commercially available electromagnetic tracking systems.

Manufacturer	Model	Tracking Distance	Position Accuracy (RMS)	Orientation Accuracy (RMS)	Average Latency	Measurement Rate
Northern Digital Inc., Waterloo, ON, Canada	Aurora-Cube Volume-5DOF [70]	N/A	0.70 mm	0.2°	N/A	40 Hz
	Aurora-Cube Volume-6DOF [70]	N/A	0.48 mm	0.3°	N/A	40 Hz
	Aurora-Dome Volume-5DOF [70]	660 mm	1.10 mm	0.2°	N/A	40 Hz
	Aurora-Dome Volume-6DOF [70]	660 mm	0.70 mm	0.3°	N/A	40 Hz
	3D Guidance trakSTAR-6DOF [71]	660 mm	1.40 mm	0.5°	N/A	80 Hz
	3D Guidance driveBAY-6DOF [71]	660 mm	1.40 mm	0.5°	N/A	80 Hz
Polhemus Inc., Colchester, VT, USA	Viper [72]	N/A	0.38 mm	0.10°	1 ms	960 Hz
	Fastrak [73]	N/A	0.76 mm	0.15°	4 ms	120 Hz
	Patriot [74]	N/A	1.52 mm	0.40°	18.5 ms	60 Hz
	Patriot Wireless [75]	N/A	7.62 mm	1.00°	20 ms	50 Hz
	Liberty [76]	N/A	0.76 mm	0.15°	3.5 ms	240 Hz
	Liberty Latus [77]	N/A	2.54 mm	0.50°	5 ms	188 Hz
	G4 [78]	N/A	2.00 mm	0.50°	<10 ms	120 Hz

**Table 3:** Summary of commercially available inertial tracking systems.

Manufacturer	Model	Static Accuracy (Roll/Pitch)	Static Accuracy (Heading)	Dynamic Accuracy (Roll/Pitch)	Dynamic Accuracy (Heading)	Average Latency	Update Rate
Xsens Technologies B.V., Enschede, The Netherlands	MTw Awinda [82]	0.5°	1.0°	0.75°	1.5°	30 ms	120 Hz
	Xsens DOT [81]	0.5°	1.0°	1.0°	2.0°	30 ms	60 Hz
	MTi-1 [83]	0.5°	N/A	N/A	N/A	N/A	100 Hz
	MTi-2 [83]	0.5°	N/A	0.8°	N/A	N/A	100 Hz
	MTi-3 [83]	0.5°	N/A	0.8°	2.0°	N/A	100 Hz
	MTi-7 [83]	0.5°	N/A	0.5°	1.5°	N/A	100 Hz
	MTi-8 [83]	0.5°	N/A	0.5°	1.0°	N/A	100 Hz
	MTi-20 [84]	0.2°	N/A	0.5°	N/A	N/A	N/A
	MTi-30 [84]	0.2°	N/A	0.5°	1.0°	N/A	N/A
	MTi-200 [84]	0.2°	N/A	0.3°	N/A	<10 ms	N/A
	MTi-300 [84]	0.2°	N/A	0.3°	1.0°	<10 ms	N/A
	MTi-710 [84]	0.2°	N/A	0.3°	0.8°	<10 ms	400 Hz
	MTi-610 [85]	N/A	N/A	N/A	N/A	N/A	400 Hz
	MTi-620 [85]	0.2°	N/A	0.25°	N/A	N/A	400 Hz
	MTi-630 [85]	0.2°	N/A	0.25°	1.0°	N/A	400 Hz
	MTi-670 [85]	0.2°	N/A	0.25°	0.8°	N/A	400 Hz
	MTi-680 [85]	0.2°	N/A	0.25°	0.5°	N/A	400 Hz
STT Systems, Donostia-San Sebastian, Spain	iSen system [86]	N/A	N/A	<0.5°	<2.0°	N/A	400 Hz
VectorNav Technologies, Dallas, TX, USA	VN-100 [87]	0.5°	N/A	1.0°	2.0°	N/A	800 Hz
	VN-110 [88]	0.05°	N/A	N/A	2.0°	N/A	800 Hz
	VN-200 [89]	0.5°	2.0°	0.2°, 1σ	0.03°, 1σ	N/A	800 Hz
	VN-210 [90]	0.05°	2.0°	0.015°, 1σ	0.05–0.1°, 1σ	N/A	800 Hz
	VN-300 [91]	0.5°	2.0°	0.03°, 1σ	0.2°, 1σ	N/A	400 Hz
	VN-310 [92]	0.05°	2.0°	0.015°, 1σ	0.05–0.1°, 1σ	N/A	800 Hz
Advanced Navigation, Sydney, Australia	Motus [93]	0.05°	0.8°	N/A	N/A	N/A	1000 Hz
	Orientus [94]	0.2°	0.8°	0.6°	1.0°	0.3 ms	1000 Hz
	Boreas D90 [95]	0.005° *	0.006° *	N/A	N/A	N/A	1000 Hz
	Spatial FOG Dual [96]	0.005° *	0.007°	N/A	N/A	N/A	1000 Hz
	Certus Evo [97]	0.01° *	0.01°	N/A	N/A	N/A	1000 Hz
	Certus [98]	0.03° *	0.06°	N/A	N/A	N/A	1000 Hz
	Spatial [99]	0.04° *	0.08°	N/A	N/A	0.4 ms	1000 Hz
	GNSS Compass [100]	0.4°	0.4°	N/A	N/A	N/A	200 Hz
Inertial Labs, Paeonian Springs, VA, USA	KERNEL-100 [101]	0.05°	0.08°	N/A	N/A	<1 ms	2000 Hz
	KERNEL-110 [102]	0.05°	0.08°	N/A	N/A	<1 ms	2000 Hz
	KERNEL-120 [102]	0.05°	0.08°	N/A	N/A	<1 ms	2000 Hz
	KERNEL-210 [103]	0.05°	0.08°	N/A	N/A	<1 ms	2000 Hz
	KERNEL-220 [103]	0.05°	0.08°	N/A	N/A	<1 ms	2000 Hz
	IMU-P [104]	0.05°	0.08°	N/A	N/A	<1 ms	2000 Hz

**Table 5:** U.S. Food and Drug Administration approved US/MRI fusion system [147].

System Type	Manufacturer	Year of FDA Approval	US Image Acquisition	Tracking Principle	Biopsy Route
UroNav	Philips	2005	Manual sweep	Electromagnetic tracking	Transrectal
Artemis	Eigen	2008	Manual rotation	Mechanical arm	Transrectal
Urostation	Koelis	2010	Automatic US probe rotation	Real-time registration	Transrectal
HI-RVS	Hitachi	2010	Real-time biplanar transrectal US	Electromagnetic tracking	Transrectal or transperineal
GeoScan	BioJet	2012	Manual sweep	Mechanical arm	Transrectal or transperineal

**Table 6:** A summary of US image fusion applications.

Reference	Modality for Fusion	Tracking Principle	Application
Park et al. [148]	Liver CT or MRI	Electromagnetic tracking	Biopsy of focal hepatic lesions with poor conspicuity on conventional B-mode US image
Lee et al. [149]	Liver CT or MRI	Electromagnetic tracking	Lesion detection of small hepatocellular carcinomas (HCCs)
Song et al. [150]	Liver CT or MRI	Plane registration and point registration	Improve sonographic conspicuity of HCC and feasibility of percutaneous radiofrequency ablation for HCCs not visible on conventional US images
Helck et al. [151]	Renal CT or MRI	Electromagnetic tracking	Identifiability and assessment of the dignity of renal lesions
Andersson et al. [152]	Renal CT	Electromagnetic tracking	Image-guided percutaneous radiofrequency ablation of small renal masses
Zhang et al. [153]	Pancreatic CT	Real-time registration	Image-guided percutaneous catheter drainage in treatment of acute pancreatitis
Klauser et al. [143]	Musculoskeletal CT	Internal landmarks	Image-guided sacroiliac joint injection
Lee et al. [141]	Thigh MRI	Real-time registration	Selecting the appropriate biopsy site in patients with suspected myopathies
Rubenthaler et al. [154]	Renal MRI/contrast enhanced US	Electromagnetic tracking	Classification of unclear and difficult renal lesions

## Abbreviations

The following abbreviations are used in this manuscript:

2D	Two dimensions
3D	Three dimensions
CCD	Charge-coupled device
Corp.	Corporation
CT	Computed tomography
DOF	Degree-of-freedom
ECIRS	Endoscopic combined intrarenal surgery
FDA	Food and drug administration
FOV	Field of view
FPS	Frames per second
GPU	Graphics processing unit
HCC	Hepatocellular carcinoma
HDR	High-dose-rate
IMU	Inertial measurement unit
Inc.	Incorporated
LED	Light emitting diode
MEMS	Microelectromechanical system
MP	Megapixel
MRI	Magnetic resonance imaging
NDI	Northern Digital Inc.
PCNL	Percutaneous nephrolithotomy
PET	Positron emission tomography
RMS	Root mean square
RVS	Real-time virtual sonography
TDOA	Time difference of arrival
TDOF	Time difference of flight
TOA	Time of arrival
TOF	Time of flight
TRUS	Transrectal ultrasound
US	Ultrasound
USB	Universal serial bus
